# MicroRNA-181c targets Bcl-2 and regulates mitochondrial morphology in myocardial cells

**DOI:** 10.1111/jcmm.12563

**Published:** 2015-04-20

**Authors:** Hongjiang Wang, Jing Li, Hongjie Chi, Fan Zhang, Xiaoming Zhu, Jun Cai, Xinchun Yang

**Affiliations:** Department of Cardiology, Beijing Chaoyang Hospital, Capital Medical UniversityBeijing, China

**Keywords:** microRNA, bcl-2, mitochondria, apoptosis

## Abstract

Apoptosis is an important mechanism for the development of heart failure. Mitochondria are central to the execution of apoptosis in the intrinsic pathway. The main regulator of mitochondrial pathway of apoptosis is Bcl-2 family which includes pro- and anti-apoptotic proteins. MicroRNAs are small noncoding RNA molecules that regulate gene expression by inhibiting mRNA translation and/or inducing mRNA degradation. It has been proposed that microRNAs play critical roles in the cardiovascular physiology and pathogenesis of cardiovascular diseases. Our previous study has found that microRNA-181c, a miRNA expressed in the myocardial cells, plays an important role in the development of heart failure. With bioinformatics analysis, we predicted that miR-181c could target the 3′ untranslated region of Bcl-2, one of the anti-apoptotic members of the Bcl-2 family. Thus, we have suggested that miR-181c was involved in regulation of Bcl-2. In this study, we investigated this hypothesis using the Dual-Luciferase Reporter Assay System. Cultured myocardial cells were transfected with the mimic or inhibitor of miR-181c. We found that the level of miR-181c was inversely correlated with the Bcl-2 protein level and that transfection of myocardial cells with the mimic or inhibitor of miR-181c resulted in significant changes in the levels of caspases, Bcl-2 and cytochrome C in these cells. The increased level of Bcl-2 caused by the decrease in miR-181c protected mitochondrial morphology from the tumour necrosis factor alpha-induced apoptosis.

## Introduction

Mitochondria play important roles in many cellular processes, such as supplying energy, signalling, cellular differentiation and growth 1,2. Mitochondria also play a leading role in the decision between cell death and cell survival through the control of signalling to induce apoptosis. Recent work indicates that the members of Bcl-2 family play a key role in the control of mitochondrial membrane permeability, fission and fusion 3, as well as cellular homoeostasis related to metabolism, autophagy and endoplasmic reticulum function 4. There are a total of 25 pro- and anti-apoptotic proteins in the Bcl-2 family. It was reported that the level of the key anti-apoptotic protein, Bcl-2, was decreased after myocardial ischaemia-reperfusion (I/R) 5, and overexpression of pro-survival Bcl-2 family proteins protected against myocardial I/R injury and attenuated apoptosis 6. Cardio-specific overexpression of Bcl-2 contributed to the cardioprotection 7.

MicroRNAs (miRNAs) are small non-coding RNAs of 21-25 nucleotides that negatively modulate gene expression at the post-transcriptional level. The regulation by miRNAs is achieved *via* its incomplete or complete complementary binding to target sequences within the 3′ untranslated region (UTR) of mRNA^8^. The fate of target mRNAs is primarily dependent on its complementarity to the miRNA. The mature miRNA guides the RNA-induced silencing complex (RISC), the cytoplasmic effector molecule in RNA interference, to the mRNA target sequence. These mature miRNA:RISC complexes reduce protein expression 9. Several groups have proposed that miRNAs play critical roles in cardiovascular physiology and the pathogenesis of cardiovascular diseases 10–12. Cardioprotective interventions, such as ischaemic preconditioning, could induce the changes in miRNAs 13,14. MiR-181c plays an important role in the inflammatory response and energy metabolism. Androulidaki *et al*. 15 found that miR-181c regulated Akt1, and was involved in the lipopolysaccharide-induced macrophage inflammatory reaction. However, no study has been conducted to investigate the direct relationship between the mitochondrial morphology in myocardial cells and miR-181c.

The mitochondrial morphology is closely related to apoptosis in myocardial cells. Apoptosis plays a key role in many cardiovascular diseases, and is important for the maintenance of cardiomyocyte homoeostasis. Studies have discovered the presence of miRNAs in the mitochondria of liver cells, Hela cells and human myoblasts 16–19. MiR-181c is proposed to be involved in regulating cardiomyocyte apoptosis. However, the relationship between the Bcl-2 family and miR-181c is not fully understood. The goal of this study was to determine whether miRNAs could be translocated into the mitochondria and investigate the possible pathophysiological implications in cardiac myocytes.

## Materials and methods

### Plasmid construction

The EGFP reporter vector pcDNA3/EGFP containing miR-binding sites was constructed as described previously 20. In brief, the mouse Bcl-2 3′UTR sequence containing putative miR-181c target site was inserted between the BamHI and EcoRI sites in pcDNA3. Similarly, 3′UTR mutants, which contain mutated Bcl-2 binding sites, were cloned into pcDNA3/EGFP at the same location. The substituted nucleotides in the mutants were selected to avoid producing binding sites for other miRNAs. All constructs were verified by sequencing. The primer sets used to generate 3′UTR fragments were shown in Table1.

**Table 1:** The primer sets used to generate specific 3′UTR fragments

3′UTR	Primer
type (WT)	
BCL2-WT-top	GATCCAGAGAGAATAAAAAGTTTCAG**GAATGT**ATG**GAATGT**GGAGG*
BCL2-WT-bot	AATTCCTCC**ACATTC**CAT**ACATTC**CTGAAACTTTTTATTCTCTCTG*
Seed mutant (SM)	
BCL2-SM-top	GATCCAGAGAGAATAAAAAGTTTCAG**CTTACA**ATG**CTTACA**GGAGG*
BCL2-SM-bot	AATTC CTCC**TGTAAG**CAT**TGTAAG**CTGAAACTTTTTATTCTCTCTG*
miR-181c mimic	AACAUUCAACCUGUCGGUGAGU
ASO-miR-181c	ACUCACCGACAGGUUGAAUGUU
Ctrl ASO	UGACUGUACUGAGACUCGACUG
Ctrl mimics	UUCUCCGAACGUGUCACGUTT
	ACGUGACACGUUCGGAGAATT

* Bold marked as target sites, the underlined marked as restriction sites.

### EGFP reporter assay

NIH3T3 cells were transfected with pcDNA3/EGFP-Bcl-2 UTR, pcDNA3/EGFP-M-Bcl-2 UTR, control vector, miR-181c mimic, control mimic, miR-181c antisense oligonucleotide (ASO), control ASO, or the reporter plasmids described above in 24-well plates. An expression vector pDsRed-C expressing red fluorescent protein (RFP) was used for normalization. The cells were lysed with RIPA Lysis Buffer (50 mM TrispH 7.4, 150 mM NaCl, 1% NP-40, 0.5% sodium deoxycholate and 0.1% SDS) at 48 hrs post-transfection.

The activities of EGFP and RFP in the supernatant were assayed using the Dual-Luciferase Reporter Assay system. Results were expressed as relative fluorescence activities by normalizing to the pDsRed-C activity. The activities of EGFP and RFP of the empty control vector and the corresponding mutant reporter vector were used for comparison. All assays were performed in triplicate.

### Cell isolation and culture

All experimental procedures involved animals were approved by the Institutional Animal Care and Use Committee of Capital Medical University, Beijing, China. Ventricular myocytes were isolated from 1-day-old Kunming mice as described previously 21. The isolated hearts were placed in ice-cold Hanks balanced salt solution (HBSS). The apex of the ventricle from the lower 1/3 of the heart was separated from the rest of the heart and cut into small pieces with surgical scissors. The heart pieces were incubated in 3 ml of digestion buffer (1 mg/ml pancreatin and 0.75 mg/ml collagenase in HBSS), and gently stirred at 37°C for 10 min. The digested tissue was collected in 5 ml of high glucose DMEM supplemented with 20% heat-inactivated foetal bovine serum (FBS). The digestion process was repeated six times. The cells were then centrifuged at 283 × g for 5 min. and resuspended in serum-containing media (high glucose DMEM with 10% FBS and 1% penicillin-streptomycin). The cells were pre-plated in 100 mm TC-treated culture dishes (Corning, NY, USA) for 1 hr to attach non-cardiac cells. The density of non-attached cells was counted using a hemocytometer and cells were plated into 6-well culture plates at a density of 1 × 10^6^ cells per well. After incubation for 48 hrs, the culture medium was replaced with high glucose DMEM containing 2% FBS and 5-bromo-2-deoxyuridine (100 μM).

### MiR-181c mimic and inhibitor

The miR-181c mimic and inhibitor were synthesized by Shanghai Gene Pharma Company (Shanghai, China), and their sequences were shown in Table2. After being starved in serum-free medium for 24 hrs, myocardial cells were transfected with miR-181c mimic, inhibitor or controls using Lipofectamine 2000 (Invitrogen, Carlsbad, CA, USA) according to the manufacturer’s instructions. At 6 hrs after transfection, the fluorescence of 5-carboxyfluorescein (5′FAM) could be detected with a fluorescence microscope. Twenty-four hours after transfection, the myocytes were harvested and used for expression analysis of Bcl-2, caspase-3, cytochrome c (Cyto-c), tumour necrosis factor alpha (TNF-α), miR-181c, pre-miR-181c, ND4, CYTB and GAPDH.

**Table 2:** The sequences of miR-181c mimic, inhibitor and their negative control

Name	Modify	Primer (5′–3′)
Mimic	5′FAM	AACAUUCAACCUGUCGGUGAGU
		UCACCGACAGGUUGAAUGUUUU
Inhibitor	5′FAM	CAGUACUUUUGUGUAGUACAA
Mimic negative control (NC)	Sense	UUCUCCGAACGUGUCACGUTT
	Anti-sense	ACGUGACACGUUCGGAGAATT
Inhibitor NC		ACUCACCGCAGGUUGAAUGUU

### Evaluation of transfection efficiency for miR-181c mimic and inhibitor

The myocardial cells were seeded in 96-well flat clear bottom black polystyrene TC-treated microplates (Corning® #3603). After being transfected with miR-181c mimic or its inhibitor at 37°C for 6 hrs, the cells were stained with the membrane probe Dil (7 μg/ml; Beyotime, Haimen, China) at 37°C for 15–20 min. for cell number counting. To evaluate transfection efficiency for miR-181c mimic and inhibitor, the fluorescence intensity was quantified with the Thermo Fisher Scientific Cellomics ArrayScan™ Vti (Thermo Fisher Scientific Inc. Waltham, MA, USA) automated fluorescent microscopic imaging system designed for high content screening of both fixed and live cells. Luciferase reporter assays were performed on duplicate plates and the reporter expression was normalized using cell numbers.

### TNF-α treatment

Mouse myocardial cells were transfected with miR-181c mimic or inhibitor. At 6 hrs after transfection, cells were treated with TNF-α (10 ng/ml) for 1 hr. At the end of treatment, the cells were harvested for total RNA and protein isolation.

### Isolation of mitochondria by magnetic antibody cell sorting method

Mitochondria were isolated from mouse myocardial cells with a mitochondria isolation kit (Miltenyi, Cologne, Germany). The isolates mitochondria was performed with supramagnetic microbeads conjugated with a monoclonal antibody that specifically binds to the translocase protein of the outer mitochondrial membrane 22 (TOM22) 22. Briefly, myocardial cells were washed with ice-cold PBS and scraped. Then the cells were resuspended in 1 ml of lysis buffer supplemented with a EDTA-free protease inhibitor cocktail (Roche, Basel, Switzerland). Fifty μL of anti-TOM22 microbeads was added to the magnetically labelled mitochondria. The mixture was incubated for 1 hr in the refrigerator (2–8°C) with gentle shaking. The mixture of cell lysate and magnetic beads was loaded onto a column placed in a magnetic field separator. The magnetically labelled mitochondria were retained in the column during washing. The column was removed from the separator, and the magnetically labelled mitochondria were flushed out. The mitochondria were collected by centrifugation at 13,000 × g for 2 min. at 4°C, and the supernatant was discarded. The total mitochondrial RNA (mtRNA) was isolated with TRIzol for RT-qPCR analysis. An aliquot of mitochondria pellets was re-suspended in 100 ml of storage buffer (magnetic antibody cell sorting) for transmission electronic microscopy and western blotting analyses.

### Transmission electron microscopy analysis

Mitochondria were checked with transmission electron microscopy (TEM) to ensure their integrity. After fixation with aldehyde mixture, the myocardia cells were post-fixed in 1% osmium tetroxide at pH 7.2 and 0.1 M cacodylate at 4°C for 2 hrs, gradually dehydrated in ethanol (30–100%), and embedded in Epon-812. Ultrathin sections were prepared with a diamond knife (Diatom, Bienne, Switzerland) on an ultramicrotome (Raichert-Nissei, Tokyo, Japan). Thin sections (70 nm) were collected onto 200 mesh cooper grids, and examined with a Hitachi H-7100 transmission electron microscope (Hitachi, Tokyo, Japan) after being stained with gold in immunogold labelling solution.

### Reverse transcription quantitative real-time polymerase chain reaction

Total RNA was isolated with TRIzol® (Invitrogen). Reverse transcription was performed with MMLV reverse transcriptase (Epicentre, Madison, WI, USA) according to the manufacturer’s instructions. SYBR-green based quantitative PCR (qPCR) was performed on an ABI PRISM 7500 system (Applied Biosystems, Foster City, CA, USA). The expression levels of miR-181c, pre-miR-181c and other selected RNAs (Bcl-2, caspase-3, Cyto-c, TNF-α, miR-181c, ND4, CYTB, and GAPDH) were normalized using 16S ribosomal RNA (rRNA; for mitochondrial fractions) 1 and β-actin (for whole cell) respectively. The PCR experiments were repeated three times, each using separate sets of samples. The RT stem-loop primers, PCR primers, and the probes were shown in Table3.

**Table 3:** The RT stem-loop primers, the PCR primers and the probes

Name	Primer (5′–3′)
mmu-miR-181cRT primer	GCGCGTGAGCAGGCTGGAGAAATTAACCACGCGCACTCAC
mmu-miR-181cF	GCAACATTCAACCTGTC
Reverse primerR	GAG CAG GCT GGA GAA
mmu-pre-181cF	CAAGGGTTTGGGGGAACA
mmu-pre-181cR	GGGTCCACTCAACGGTCG
mCaspase3F	GCTGGACTGTGGCATTGAGA
mCaspase3R	GACTGGATGAACCACGACCC
mBcl2F	CTACCGTCGTGACTTCGCAG
mBcl2R	TCTCCCTGTTGACGCTCTCC
mCyto-CF	GTTCGGGCGGAAGACAGG
mCyto-CR	CTGCCCTTTCTCCCTTCTTC
mTNFa F	CCCTCCAGAAAAGACACCATG
mTNFa R	CACCCCGAAGTTCAGTAGACAG
mGAPDHF	GTGCCGCCTGGAGAAACC
mGAPDHR	GGTGGAAGAGTGGGAGTTGC
mβ-actin F	AGAGGGAAATCGTGCGTGAC
mβ-actin R	AACCGCTCGTTGCCAATAGT
mND4F	AAAATCACTAATCGCCTACTCCTC
mND4R	CCATGATTATAGTACGGCTGTGG
mCYTBF	CGCAGTCATAGCCACAGCAT
mCYTBR	AAGCGAAGAATCGGGTCAAG
16S RNA forward	TGCCTGCCCAGTGACTAAAGT
16SRNAreverse	AACAAGTGATTATGCTACCTTTGCA

### Western blot

Western blot was performed as previously described 23. Briefly, equal amounts (30 μg) of proteins were separated on a polyacrylamide gel, and electro-transferred to polyvinylidene fluoride membranes (Millipore Corp., Billerica, MA, USA). Membranes were blocked overnight with the primary antibody against Bcl-2 (1:1000 dilution, #2876; Cell Signaling Technology, Beverly, MA, USA), Caspase-3 (1:1000 dilution, #9662; Cell Signaling Technology), Cytochrome C (1:1000 dilution, #4272; Cell Signaling Technology) or GAPDH (1:2000 dilution; ZSGB-BIO, Beijing, China). After washing, membranes were incubated with goat anti-rabbit antibody (1:1000 dilution; ZSGB-BIO). Immunoreactive bands were visualized using the BIO-RAD ChemiDoc XRS imaging system according to the manufacturer’s instruction. Densitometric analysis was performed with Quantity One software (Bio-Rad, Shanghai, China). GAPDH was used for normalization.

### Statistical analysis

All experiments except western blot were performed in triplicates. The results were expressed as means ± SD. The comparative CT method was applied in the quantitative real-time RT-PCR analysis. All statistical analyses were performed with SPSS version 13.0. The data were analysed with Student’s *t*-test or one-way anova, and *P* < 0.05 was considered statistically significant.

## Results

### miR-181c targeted the 3′UTRs of Bcl-2

**Figure 1:**
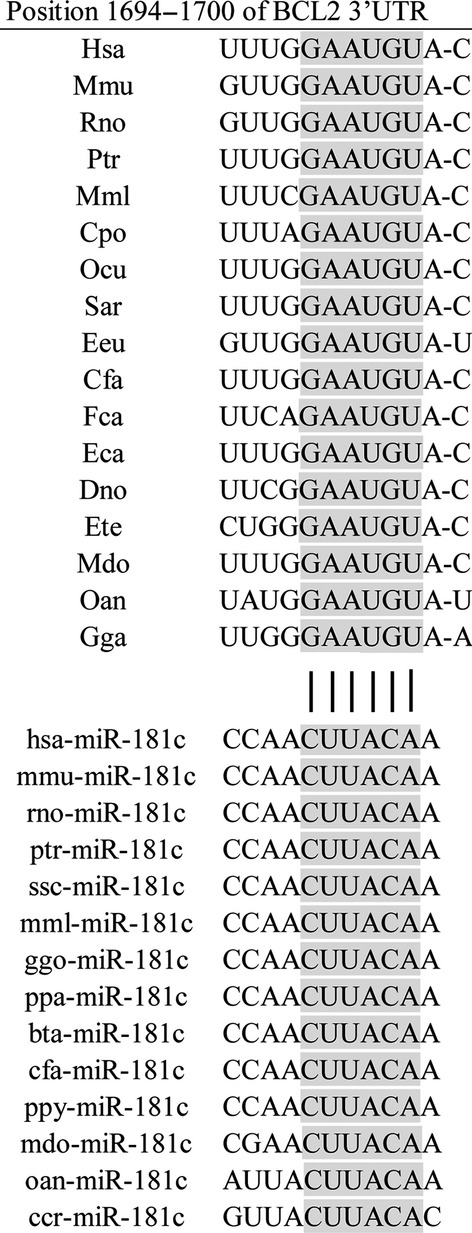
The seed sequences of Bcl-2 3′UTRs targeted by miR-181c are highly conserved across species (from TargetScan). With the computational miRNA target prediction algorithms at TargetScan, Bcl-2 was identified as one of potential targets of miR-181c.

**Figure 2:**
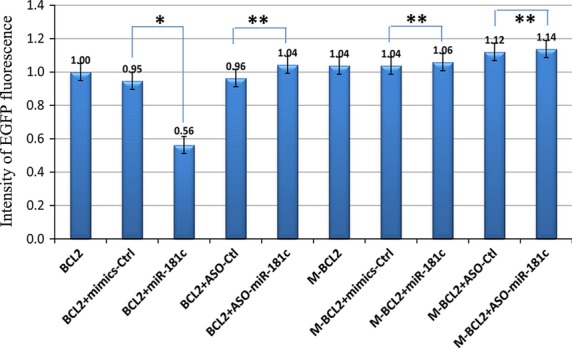
EGFP Reporter Assay. The intensity of EGFP fluorescence in NIH3T3 cells transfected with pcDNA3/EGFP-Bcl2 3′UTR and miR-181c mimic reduce the luciferase activity compared with the control group. Luciferase assays were performed three times in triplicate. (*P < 0.05; **P > 0.05)

Using the computational miRNA target prediction algorithms at TargetScan (http://targetscan.org, Release 6.2), Bcl-2 was identified as one of the potential targets of miR-181c (Fig.1). The common names corresponding to the scientific names in the figure were listed in Table4. The seed sequences of the 3′UTRs targeted by miR-181c are highly conserved (Fig.1), which may be critical in the normal physiology. The mutated seed sequences of the 3′UTR of Bcl-2 were shown in Table1 to exclude off-target effects. To determine whether the interaction between miR-181c and Bcl-2 mRNA is direct, an EGFP reporter vector was constructed in which 3′UTR fragment of Bcl-2 containing putative target sites was cloned down-stream of the EGFP coding sequence. An RFP reporter with either a miR-181c vector or ASO was cotransfected into NIH3T3 cells. As shown in Figure2, the intensity of EGFP fluorescence in NIH3T3 cells transfected with pcDNA3/EGFP-Bcl2 3′UTR and miR-181c mimic was decreased by 39% as compared with that of the control group. Inhibition of miR-181c by ASO had no significant influence on EGFP expression of the reporter vector containing the 3′UTR of Bcl-2. Importantly, EGFP expression in the mutated reporter vector was not affected by miR-181c overexpression or inhibition, highlighting the importance of the miR-181c binding site in the regulation. These results show that miR-181c targets Bcl-2 by directly binding to its 3′UTR.

**Table 4:** List of scientific and common names

	Scientific name	Common name
Mmu	Musmusculus	House mouse
Rno	Rattusnorvegicus	Rat
Hsa	Homo sapiens	Human
Ptr	Pan Troglodytes	Chimpanzee
Mml	Macacamulatta	Rhesus macaque
Cpo	CambarellusPatzcuarensis (var. orange)	Orange dwarf crayfish
Ocu	Oncorhynchus	Salmon
Sar	Scatophagusargus	Spotted scat (fish)
Eeu	Euonymus europaeus	European spindle tree
Cfa	CannisFamiliaris	Dog
Eca	EqqusCaballus	Horse
Bta	Bostaurus	Oxen
Dno	Dendragapusobscurus	Grouse
Laf	Laruscalifornicus	California gull
Ete	Embiotocidae	Surf perch
Mdo	Monodelphisdomestica	Grey short-tailed opossum
Fca	Feliscatus	Cat
Oan	Ornithorhynchusanatinus	Platypus
Gga	Gallus gallus	Chicken

### Integrity and purity of mitochondria

**Figure 3:**
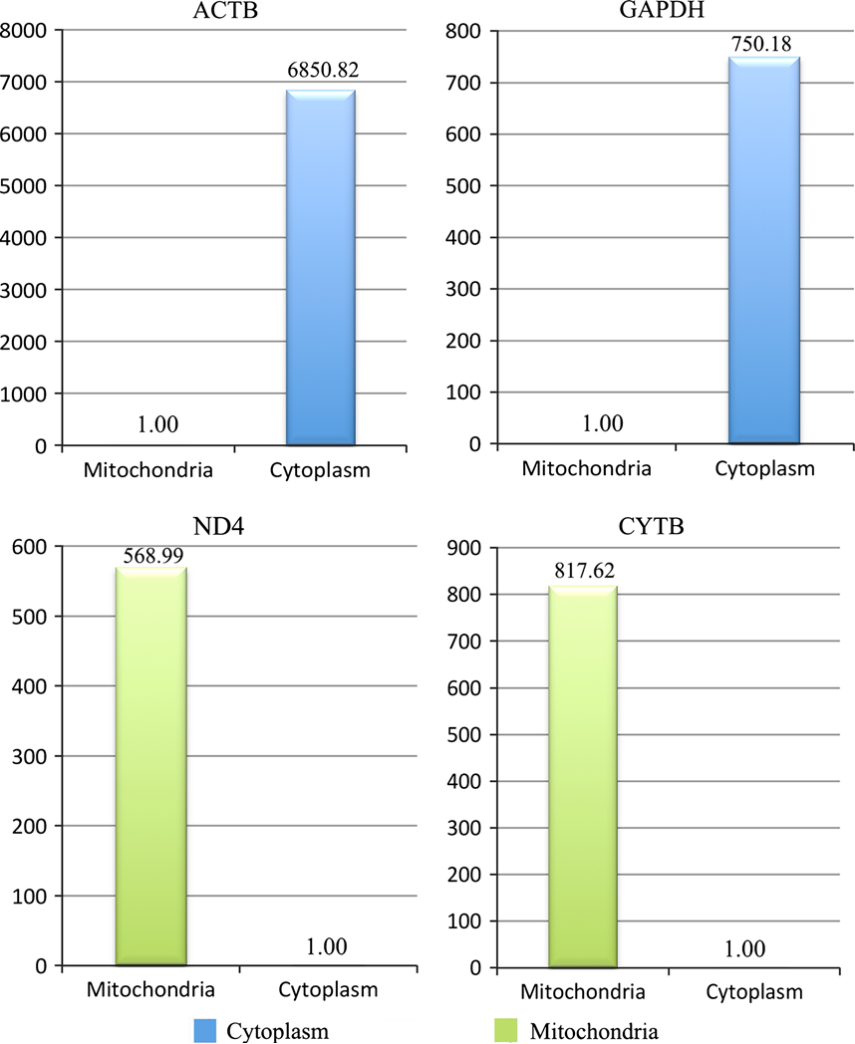
Quantification of the mitochondrial to nuclear DNA ratio in the mitochondrial fraction using two mitochondrial genes (ND4, CYTB) and two nuclear genes (ACTB, GAPDH). The relative expression of the two nuclear genes GAPDH and ACTB were respectively 1.33 × 10^−3^ to 1.46 × 10^−4^ lower in the mtRNA extract than in the cytosolic extract (A and B). The relative expression of two mitochondrial genes ND4 and CYTB were respectively 568.99 and 817.62 times greater in the mtRNA extract than in the cytosolic RNA extract (C and D). These results confirmed the high mitochondria enrichment in the mitochondrial fraction. This indicated a very low contamination of the mitochondrial fraction by the genomic mRNA.

The mitochondrial DNA to nuclear DNA ratio was determined using two mitochondrial genes (ND4, CYTB) and two nuclear genes (ACTB, GAPDH). The cytosolic GAPDH and ACTB mRNAs were absent in the mitochondrial fraction. The relative expression levels of nuclear genes GAPDH and ACTB were 1.33 × 10^−3^ and 1.46 × 10^−4^ lower, respectively, in the mtRNA extract compared to the cytosolic extract (Fig.3A and B). On the contrary, the relative expression levels of mitochondrial genes ND4 and CYTB were 568.99 and 817.62 times higher, respectively, in the mtRNA extract compared to the cytosolic RNA extract (Fig.3C and D). These results confirmed the enrichment of the mitochondrial fraction, and indicated minimal contamination of cytosolic mRNA in the mitochondrial fraction.

**Figure 4:**
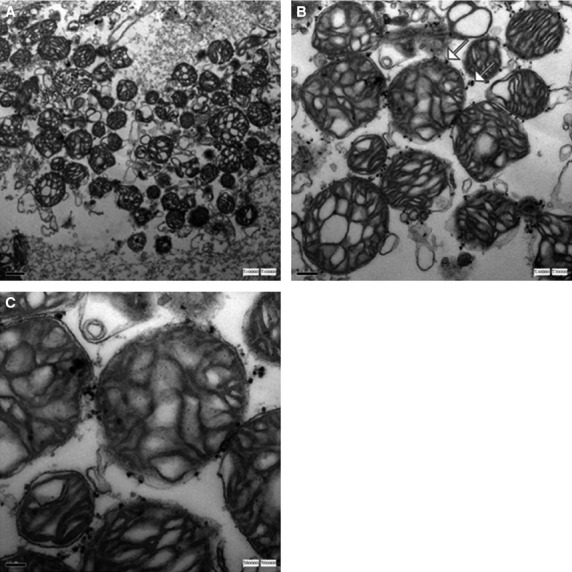
The integrity of mitochondrial membranes and ultrastructure seemed to be preserved as well as demonstrated on the TEM images. The continuous double membranes, cristae, the normal matrix density and the supramagnetic microbeads conjugated to the antibody anti-TOM22 bound to the translocase outer membrane protein were observed. TEM magnification: (A) ×10,000. (B) ×30,000. (C) ×60,000.

The integrity of mitochondrial membrane and ultrastructure was preserved as demonstrated on the TEM images (Fig.4). The continuous double membranes, cristae, and normal matrix density were clearly observed. In addition, the supramagnetic microbeads conjugated to the anti-TOM22 antibody bound to translocase. The mitochondria integrity also confirmed the minimal cytosolic RNA contamination during mitochondria preparation.

### Different concentrations of miR-181c mimic or inhibitor altered pro-survival Bcl-2 protein level in the myocardial cells

**Figure 5:**
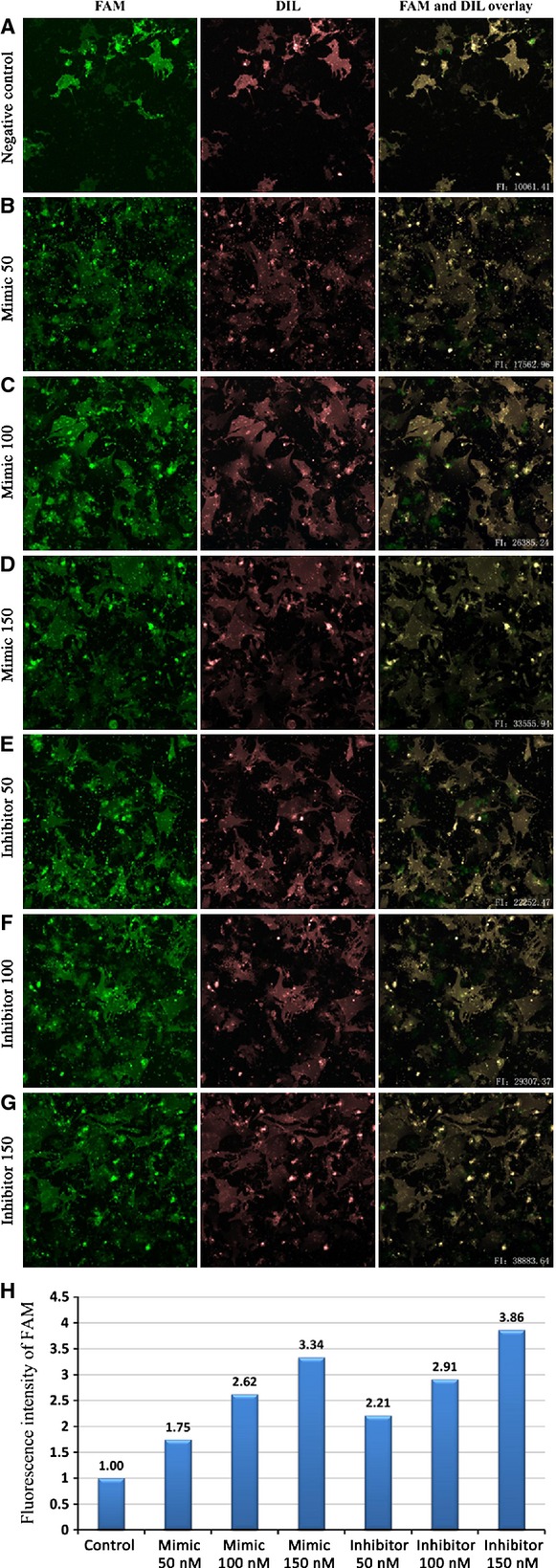
Setting cell membrane of the transfected cardiomyocytes labelled by the membrane fluorescence probe Dil as the boundary, the fluorescence intensity of FAM in the transfected cells were measured by the Cellomics ArrayScan™ Vti. FAM fluorescence (lane 1), Dil staining of cell membrane (lane 2), In the overlays (lane 3), FAM signal are visualized in green, Dil staining of cell membrane are in red. The fluorescence intensity of FAM signal was measured within the scope of the red fluorescence of Dil staining. The rows are scramble miRNA (A; negative control), mimic 50 nM (B), mimic 100 nM (C), mimic 150 nM (D), inhibitor 50 nM (E), inhibitor 50 nM (F) and inhibitor 150 nM (G). The H row show that the intracellular fluorescence intensity improved with the increase in miR-181c mimic/inhibitor concentration. These images are not scaled to the same intensity range. Fluorescence intensity was normalized by negative control intensity. FI: fluorescence intensity.

**Figure 6:**
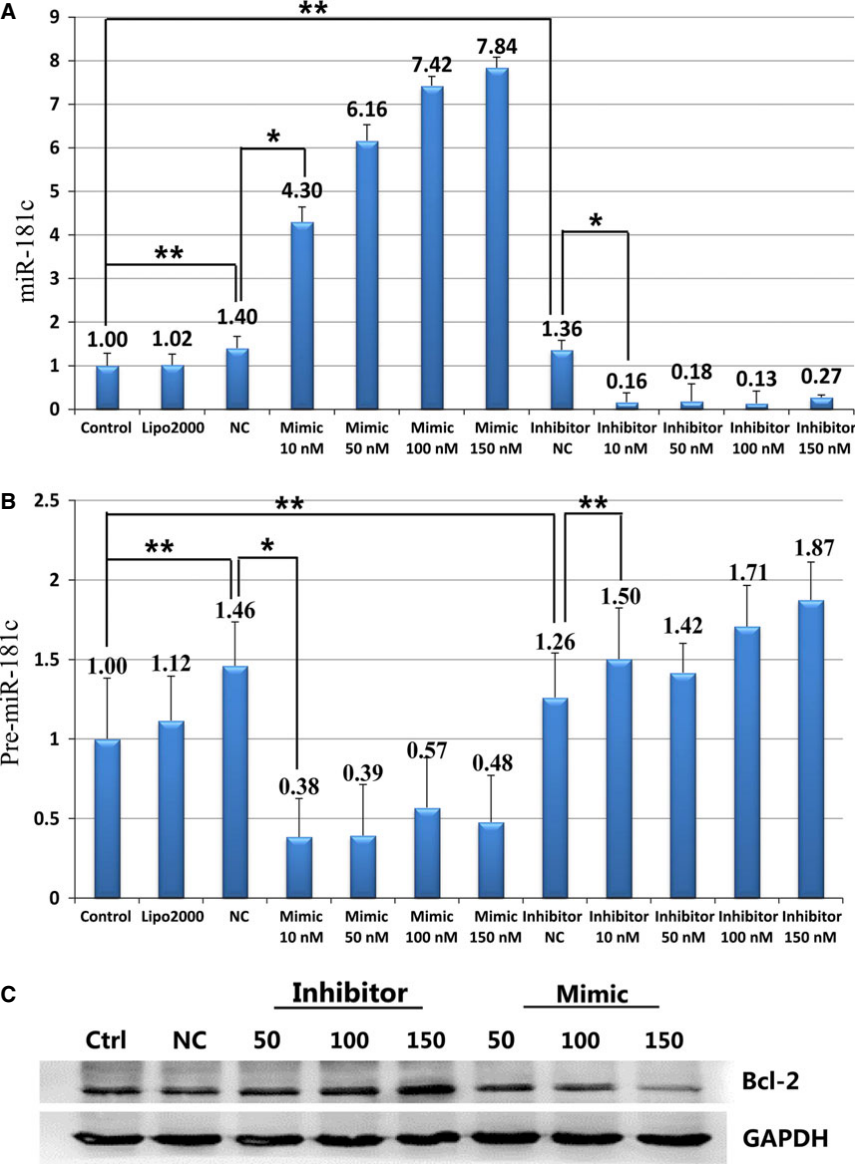
The levels of miR-181c (A) and pre-miR-181c (B) expression were titrated in myocardial cells transfected with mimic or inhibitor. Protein levels of Bcl-2 (C) changed in myocardial cells transfected with mimic or inhibitor (*P < 0.05; **P > 0.05).

Different concentrations of miR-181c mimic or inhibitor were used to treat the cells. The cell membrane of transfected cardiomyocytes was labelled with the fluorescence probe Dil. The fluorescence intensity of FAM in the transfected cells was measured with the Cellomics ArrayScanTM Vti (Fig.5A–G). With the increase in mimic/inhibitor concentrations, the intracellular fluorescence intensity was increased (Fig.5H). As determined by Dil labelling, the morphology of cardiomyocytes showed no significant changes from the usual filamentous pattern (upper panel) to a fragmented pattern with the increase in mimic/inhibitor concentrations. The levels of miR-181c and pre-miR-181c were measured with RT-PCR. Results showed a more than 4.3-fold increase in miR-181c expression (*P* < 0.05, Fig.6A) in the myocardial cells transfected with mimic, and an 84% decrease in the myocardial cells transfected with inhibitor (*P* < 0.05, Fig.6A). At the same time, the level of pre-miR-181c expression was significantly decreased in the myocardial cells transfected with mimic (*P* < 0.05, Fig.6B), while no significant change was observed in the myocardial cells transfected with inhibitor (*P* > 0.05, Fig.6B). The protein levels of GAPDH and Bcl-2 were determined by Western blot analysis after transfection of the myocardial cells with either mimic or inhibitor. Results demonstrated that Bcl-2 protein levels were decreased in the presence of mimic and were increased with the transfection of inhibitor (Fig.6C). The protein levels of Bcl-2 in myocardial cells transfected with 150 nM miR-181c mimic or inhibitor changed significantly (Fig.6C). Therefore, a lower concentration of 100 nM mimic and 100 nM inhibitor was chosen for the subsequent studies.

### miR-181c interfered with TNF-α-induced apoptosis pathway

**Figure 7:**
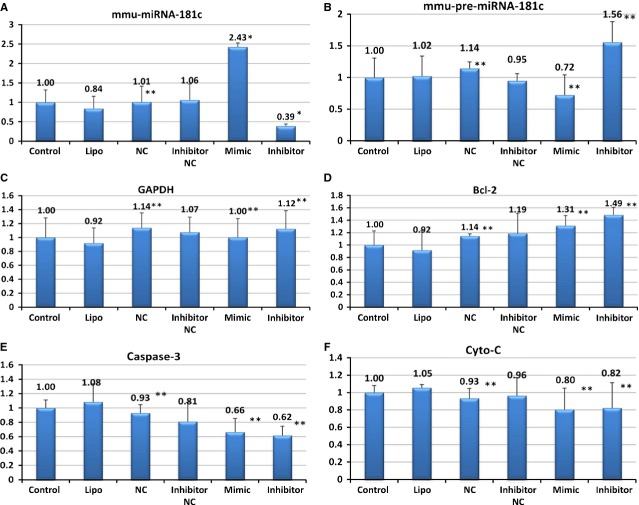
Genes included in the TNF-α-induced apoptosis pathway. The levels of miR-181c in the whole cells significantly increased after transfected with mimic (*P* < 0.05) and significantly decreased with inhibitor (*P* < 0.05) (A). While the levels of pre-miR-181c showed the trend of change, they did not reach statistical significance (*P* > 0.05) (B). The genes Bcl-2, Cyto-c and caspase-3, which are associated with the severity of heart failure and apoptosis of myocardial cells, were examined using RT-PCR. Their mRNA expression showed no significant difference (*P* > 0.05) (C–F).

Overexpression of anti-apoptotic Bcl-2 could reduce cell death and protect mitochondrial membrane potential 24. In this study, the myocardial cells were treated with TNF-α after transfection with miR-181c mimic or inhibitor. The levels of miR-181c and pre-miR-181c were examined in both whole cells and isolated mitochondria with RT-PCR. The mitochondrial gene product 16S rRNA 1 was served as the internal control. There was no detectable expression of miR-181c and pre-miR-181c in the isolated mitochondrial fraction. The level of miR-181c in whole cells was significantly increased after transfection with mimic (*P* < 0.05), and significantly decreased with inhibitor transfection (*P* < 0.05; Fig.7A). The level of pre-miR-181c showed no significant change (*P* > 0.05) after mimic or inhibitor transfection (Fig.7B). The expression levels of Bcl-2, Cyto-c and caspase-3 genes, which are associated with the severity of heart failure and apoptosis in myocardial cells, were also examined with RT-PCR. The mRNA levels of these genes showed no significant difference (*P* > 0.05) with mimic or inhibitor transfection (Fig.7C–F).

**Figure 8:**
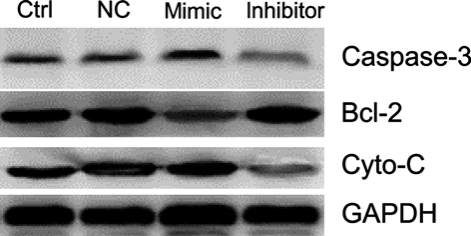
The protein levels of Bcl-2 were examined by Western blot after transfection of myocardial cells with either mimic or inhibitor. Protein levels of Bcl-2 changed significantly in transfected cells, decreasing in the presence of mimic and increasing after transfection with inhibitor (*P* < 0.05) and decreasing more than 50% with mimic (*P* < 0.05). The change in Cyto-c and caspase-3 were opposite to that of Bcl-2. The protein expression of GAPDH was no significant difference.

Bcl-2 protein levels in the myocardial cells were assessed by Western Blot after the transfection with miR-181c mimic or inhibitor. Bcl-2 protein levels were decreased by 47.8% with miR-181c mimic, and were increased by 45.5% after transfection with inhibitor (*P* < 0.05, Fig.8). The protein levels of Caspase-3 and Cyto-c increased by 37.9% and 28.2% in the presence of mimic, and decreased by 25.3% and 49.1% after transfection with inhibitor. Cyto-c and caspase-3 levels changed in an opposite way compared to Bcl-2. The protein expression of GAPDH did not change significantly.

**Figure 9:**
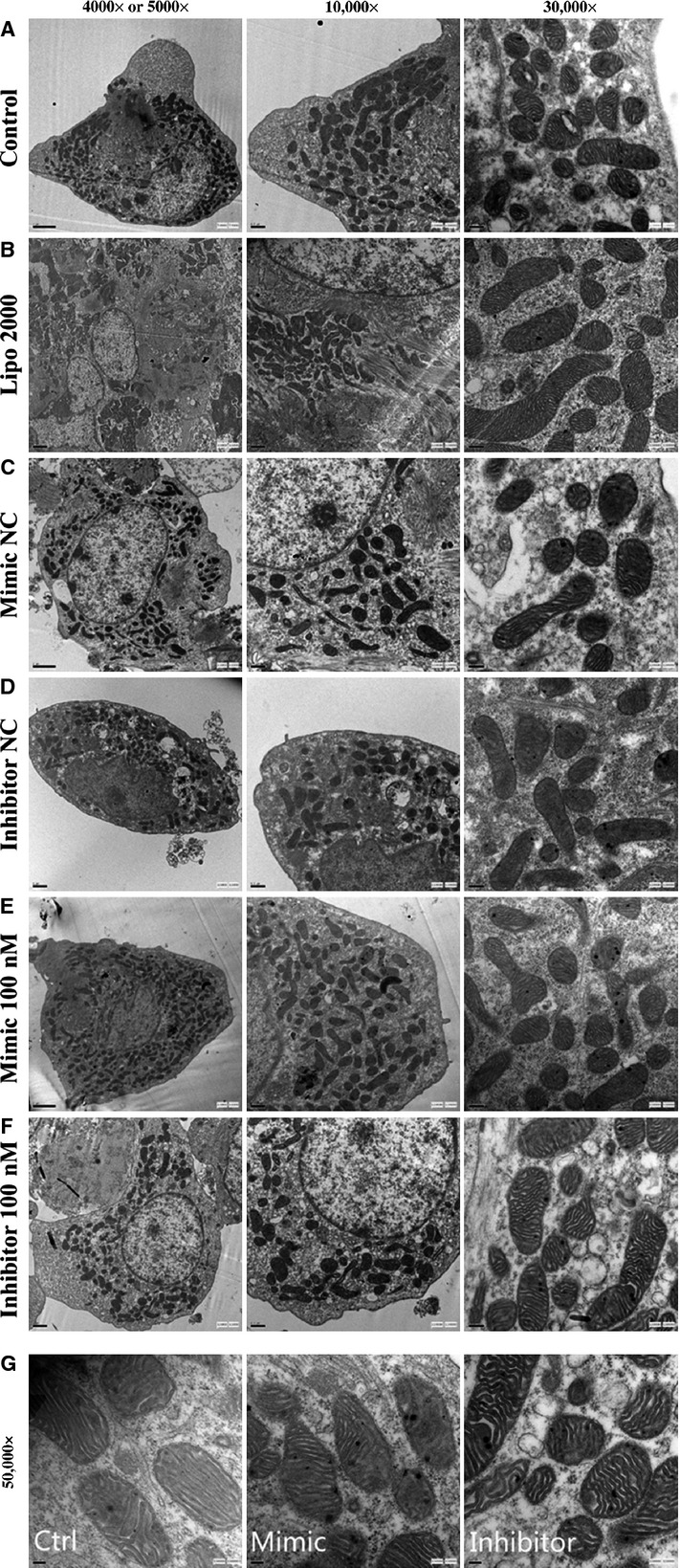
The mouse myocardial cells, transfected with miR-181c mimic/inhibitor, were intervened with TNF-α and underwent the TEM scan. There were no significant changes in the gross morphology of myocardial cells and the number of mitochondria between groups (A–F, magnification: 4000 ×  to 30000 × ). The mitochondria showed abnormal appearance such as disorganization, rupture of the double membrane and reduction or vanish of the crista after TNF-α intervention. The severity of mitochondrial damage were for mimic group>control group>inhibitor group (G, magnification: 50000 × ).

**Figure 10:**
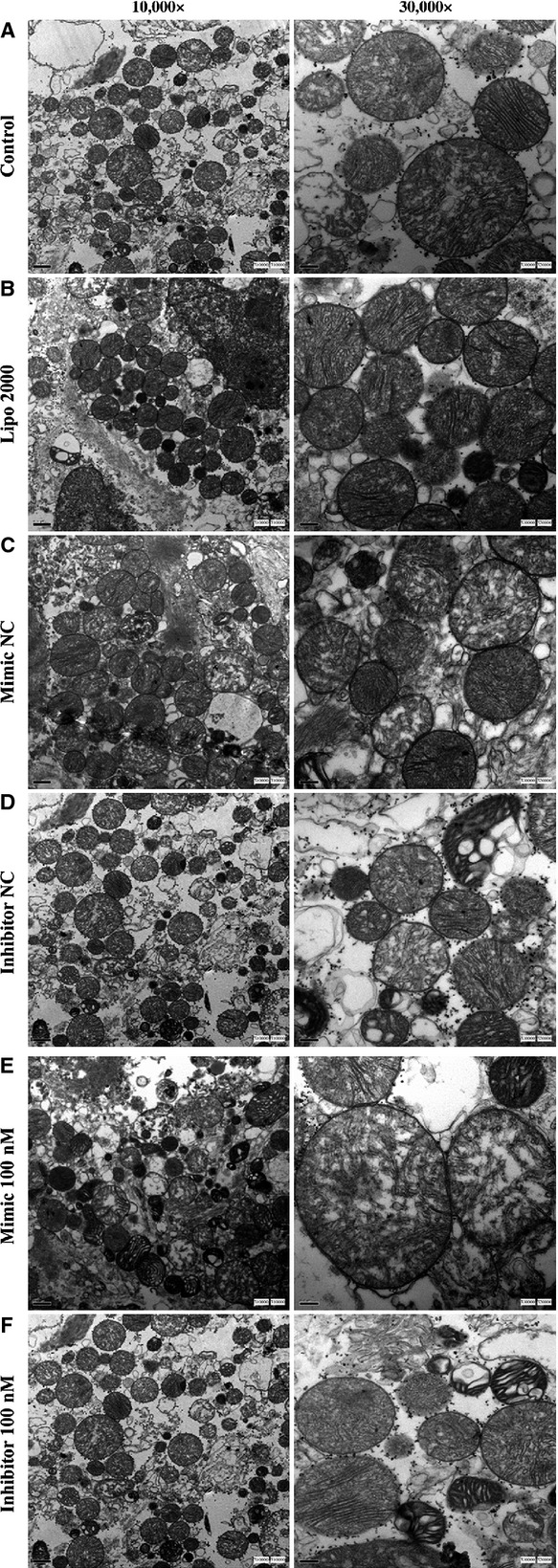
The isolated mitochondria showed the swelling of mitochondria, damaged structure of mitochondrial double membrane after TNF-α transfected. Differences in the degree of damage between groups could not be judged unambiguously on a purely qualitative basis.

At the same time, the mouse myocardial cells transfected with miR-181c mimic/inhibitor and treated with TNF-α were examined with the TEM scan. There were no significant differences in overall morphology of myocardial cells and the number of mitochondria between different groups (Fig.9A–F). The mitochondria showed abnormal features such as disorganization, rupture of the double membrane and reduction/loss of the crista after TNF-α treatment. The severity of mitochondrial damages was in the following order: mimic group>control group>inhibitor group (Fig.9G). The isolated mitochondria showed swelling and damaged structures in the double membrane after TNF-α treatment (Fig.10). However, differences in the severity of damages between different groups could not be stratified unambiguously without quantitative analysis.

## Discussion

Apoptosis is a key mechanism of cardiac myocytes loss during heart failure. It could be induced by various events such as growth factor withdrawal and toxins. The percentage of apoptotic cardiac myocytes in failing human hearts is lower (0.08–0.25%) than that in myocardial infarction where a burst of cell death occurs. However, this percentage is about 10- to 100-fold higher compared to normal hearts (0.001–0.01%) 25–27. These data suggest that enhanced apoptosis of cardiac myocyte, although at a lower level compared to myocardial infarction, results in a cumulative loss of cardiac myocytes and heart failure over time. It is controlled by many regulators, which may impose either an inhibitory effect (anti-apoptotic) or a stimulatory effect (pro-apoptotic) 28,29. On the other hand, resistance to apoptosis helps protecting heart failure.

There are two well-defined apoptotic pathways, the intrinsic/mitochondrial pathway and the extrinsic/death receptor pathway. Mitochondria play an important role in the regulation of the intrinsic apoptosis pathway. During apoptosis, the mitochondria are disrupted, resulting in smaller fragments 30–35. More than 2000 proteins are found in the mitochondria. But the majority of these proteins are derived from nuclear gene transcription and translation 36,37. There are many theories explaining how Bcl-2 proteins exert their pro- or anti-apoptotic effects. According to one theory, Bcl-2 protein activates or inactivates an inner mitochondrial permeability transition pore, which is a key factor in apoptosis through the regulation of matrix Ca^2+^, pH, and voltage. It is also possible that the pro-apoptotic members of Bcl-2 family induce the release of cytochrome c and other apoptotic factors (Smac/Diablo homologue, and Omi) into the cytosol from the mitochondria, whereas the anti-apoptotic members inhibit the releases of these factors 38. The release of cytochrome c from mitochondria results in the activation of the downstream caspase cascade, and is considered a point of no return during cell death. Overexpression of pro-survival Bcl-2 members protects cells against apoptosis induced by a variety of cytotoxic stimuli 39. Some anti-apoptotic Bcl-2 members have been shown to translocate and insert into the mitochondrial outer membrane (MOM) upon apoptotic stimulation 12,40. The pro-survival Bcl-2 proteins may inhibit MOM permeabilization by the direct interactions with the pro-apoptotic members. This complex network existing in the cytosol and the mitochondria determines the fate of the cells.

MiRNAs play a role in the regulation of DNA replication and chromosome maintenance, transcriptional activity, RNA processing, translation and stability, as well as translocation of proteins 41,42. Studies have indicated that apoptosis may also be controlled by miRNAs, which have implications in cancer 43–45, development 46,47, and various other diseases 11,48–50. MiRNAs can also sequester mRNAs for either degradation 51,52 or re-expression without *de novo* transcription 53. In our previous study, we observed that the miRNA hsa-miR-181c was significantly and differentially up-regulated in DCM samples compared with non-failing control samples 54. MiR-181c has been studied extensively in the setting of immune cell differentiation and leucemia 55–57. A previous study revealed a potential correlation between miR-181c and Bcl-2, but it did not identify the putative target sequence miR-181c in Bcl-2 55. In this study, we identified the miR-181c-targeted genes related to apoptosis using computational prediction algorithms. The direct interaction between miR-181c and Bcl-2 was confirmed using the dual-luciferase reporter assay. The impact of miR-181c on Bcl-2 was observed in primary myocardial cells. This study is the first report about the regulation of Bcl-2 by miR-181c in myocardial mitochondria. The levels of miR-181c in myocardial cells were changed with the transfection of the miR-181c mimic/inhibitor in a dose-dependent manner. The Bcl-2 protein level in myocardial cells was inversely correlated with the level of miR-181c. Previous studies not only revealed the anti-apoptotic role of Bcl-2, but also identified its role in regulating mitochondrial metabolism and function 7,58,59. In a mouse model with cardiac-specific overexpression of Bcl-2, Chen *et al*. showed that Bcl-2 protected against I/R injury and attenuated apoptosis 6. In addition, other studies provided new insights into the mechanism by which Bcl-2 mediates cardio-protection that involves altered mitochondrial adenine nucleotide metabolism 7. Although miRNAs have been discovered in mitochondria 16, no miR-181c and pre-miR-181c was detected in the mitochondria of cardiac myocyte in this study. One explanation may be that miR-181c is not trafficked across the mitochondrial membrane. The morphology of cardiomyocytes showed no significant changes with the mimic/inhibitor transfection as determined by Dil staining in this study.

Tumour necrosis factor-α induces apoptosis by more than one mechanisms, particularly by mitochondrial dependent or independent pathways, which mainly differ in the extent of caspase-8 activation 60. Activated caspase 8, in turn, mediates the cleavage of the pro-apoptotic protein Bid generating a truncated form (tBid), which will be translocated to the mitochondria. This process decreases the mitochondrial membrane potential, resulting in the release of cyto-c. Cyto-c, together with the apoptotic protease activating factor 1 (Apaf1), binds to the initiator pro-caspase 9, forming an apoptosome complex. The apoptosome complex activates other caspases including caspases 3 and 7, leading to cell apoptosis. In this study, TEM pictures showed the rupture of the double membrane, reduction or loss of the crista during TNF-α-induced apoptosis. The mitochondrial damage in the mimic group was more severe than that in the control and inhibitor groups. The mitochondrial shape was protected by down-regulating miR-181c in myocardial cells. The apoptosis-related proteins Bcl-2, Cyto-c and caspase-3 were also modulated by miR-181c during TNF-α-induced apoptosis.

In summary, Bcl-2 is a target of mouse miR-181c. The change in Bcl-2 protein was in a reverse direction with that of miR-181c. The increased level of Bcl-2 caused by the decrease in miR-181c protected mitochondrial morphology.
